# Supplementary material for: The induction of natural competence adapts staphylococcal metabolism to infection
Source: Nat Commun. 2022 Mar 21;13:1525. doi: 10.1038/s41467-022-29206-7 (PMC8938553; doi:10.1038/s41467-022-29206-7)
Supplement: Supplementary file 1 — Supplementary Information [file 41467_2022_29206_MOESM1_ESM.pdf]

Supplementary Information for

The Induction of Natural Competence Adapts Staphylococcal Metabolism to Infection

Mar Cordero[1#], Julia García-Fernández[1#], Ivan C. Acosta[1], Ana Yepes[2,3], Jose Avendano-Ortiz[4], Clivia Lisowski[3], Babett Oesterreicht[2,3], Knut Ohlsen[2,3], Eduardo Lopez-Collazo[4,5], Konrad U. Förstner[2,3,6,7], Ana Eulalio[3,8,9] and Daniel Lopez[1,2,3*]

[#] These authors contributed equally to this work

* Corresponding author: Daniel Lopez
Email: dlopez@cnb.csic.es

This PDF file includes:

Supplementary Figures 1 to 13
Supplementary Tables 1 to 5
Supplementary References

Other supplementary materials for this manuscript include the following:

Supplementary Data 1

Supplementary Figures

Supplementary Figure 1

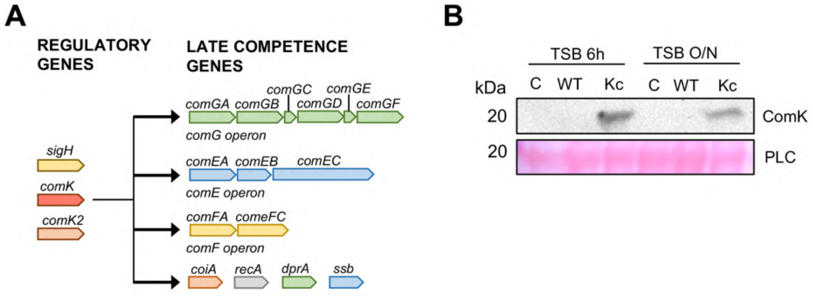

Supplementary Figure 1: The Δ*comK* mutant is more sensitive to oxidative stress than WT *Staphylococcus aureus*. A) Late competence genes and competence regulators that are present in *Staphylococcus aureus* genome, according to previous literature[1]. **B)** Immunodetection of ComK production levels in different strains during exponential (6 h) and stationary phase (O/N) of TSB cultures. *Staphylococcus aureus* produces non-detectable levels of ComK in these growth conditions. WT is wild-type strain. Kc is a strain that shows constitutive expression of *comK* irrespective of growth conditions. The strains expressed a FLAG-tagged version of ComK. C is a control unlabelled strain. The Ponceau stained nitrocellulose membrane serves as a protein loading control (PLC). These results are representative of the results obtained in three different experiments. Source data are provided as a Source Data file.

Supplementary Figure 2

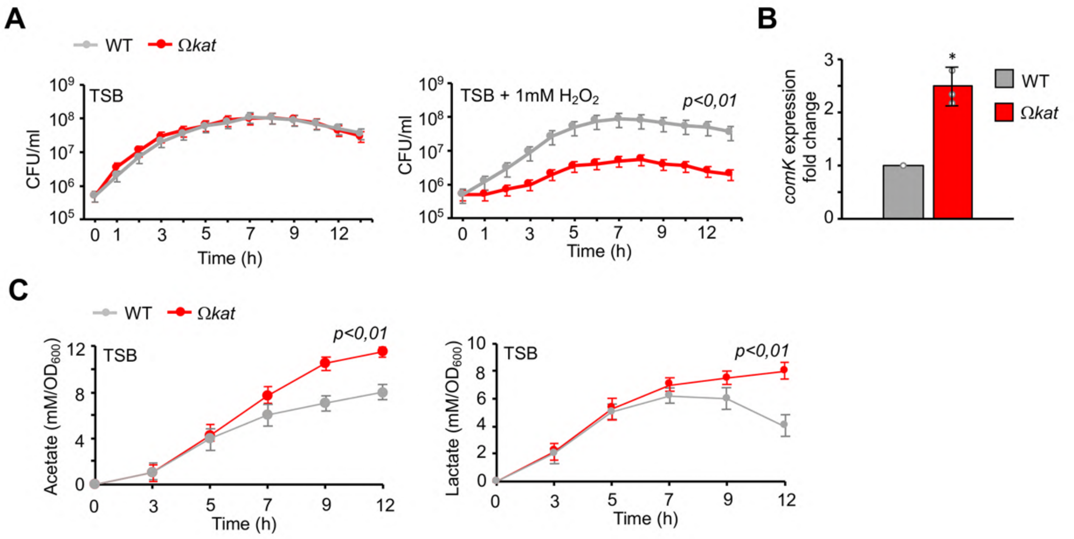

Supplementary Figure 2: A catalase-deficient mutant shows induced expression of *comK* and higher acetate and lactate production levels. A) Growth curve of WT and a catalase-deficient strain (Ω*kat*) in TSB medium in the presence or in the absence of H_2O_2 (1mM). Cultures were incubated for 12 h at 37°C with 200 rpm agitation. The Ω*kat* strain is more sensitive to oxidative damage than the WT strain. Statistical differences were measured by one-sided ANOVA with Tukey's test for multiple comparison. Data are shown as mean ± SD of three different biological replicates (n = 3). Each biological replicate included three technical replicates. **B)** qRT-PCR analysis of *comK* gene expression in WT and Ω*kat* cultures in TSB medium at exponential phase. Differences were examined by two-tailed Student's *t* test **p*<0.05. Data are shown as mean ± SD of three different biological replicates (n = 3). **C)** Determination of acetate (left panel) and lactate (right panel) levels in culture supernatants of TSB cultures. Concentration is represented in relation to culture OD_{600}. Statistical significance was measured by one-sided ANOVA with Tukey's test for multiple comparison ***p*<0.01. Data are shown as mean ± SD of three different biological replicates (n = 3). Source data are provided as a Source Data file.

Supplementary Figure 3

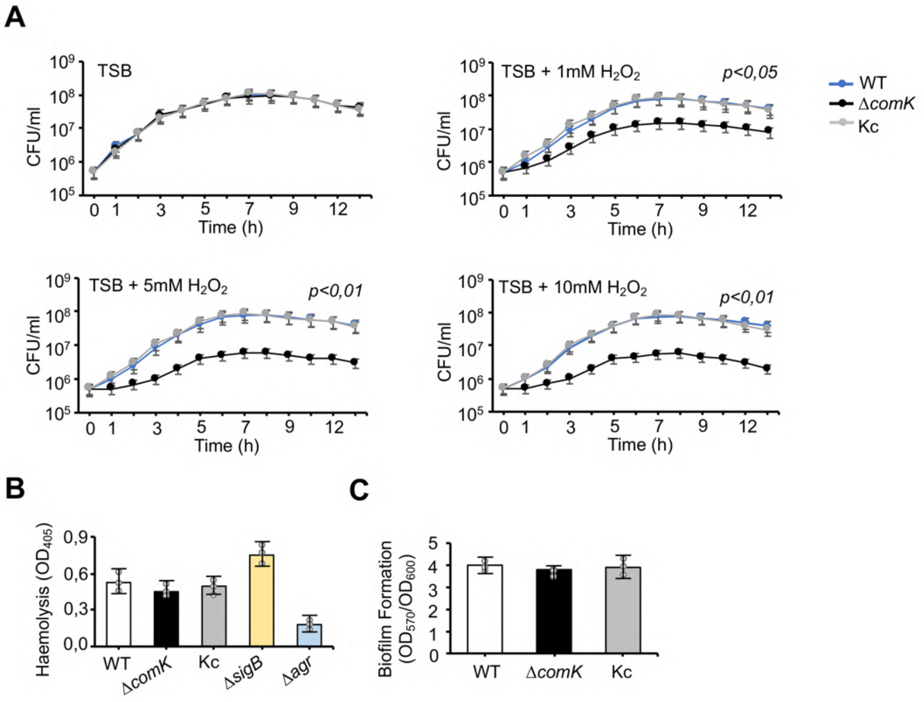

Supplementary Figure 3: The Δ*comK* mutant is more sensitive to oxidative stress than the WT strain. A) Growth curve of the WT, Δ*comK* and Kc strains in TSB medium with distinct concentrations of H_2O_2 (0-10 mM). Cultures were incubated for 12 h at 37°C with 200 rpm agitation. Statistical differences were measured by one-sided ANOVA with Tukey's test for multiple comparison. Data are shown as mean ± SD of three different biological replicates (n = 3). **B)** Haemolytic activity of WT, Δ*comK* and Kc strains. Δ*sigB* and Δ*agr* mutants represent negative and positive controls, respectively. Quantification of haemolytic activity was performed by measuring the OD_{405} of a solution of 2% erythrocytes previously incubated with the strains supernatants[2]. Significance was measured by analysis of variance one-sided ANOVA (n = 3). Data are shown as mean ± SD of three different biological replicates (n = 3). **C)** Biofilm formation in cultures grown in 24-well titer plates. Biofilm assay was performed in TSB medium supplemented with $MgCl_2$ 100 mM, as reported in the literature[3]. Biofilms were stained with crystal violet (1%) and quantified by spectrophotometry analysis[4]. Significance was measured by analysis of variance one-sided ANOVA. Data are shown as mean ± SD of three different biological replicates (n = 3). Source data are provided as a Source Data file.

Supplementary Figure 4

A

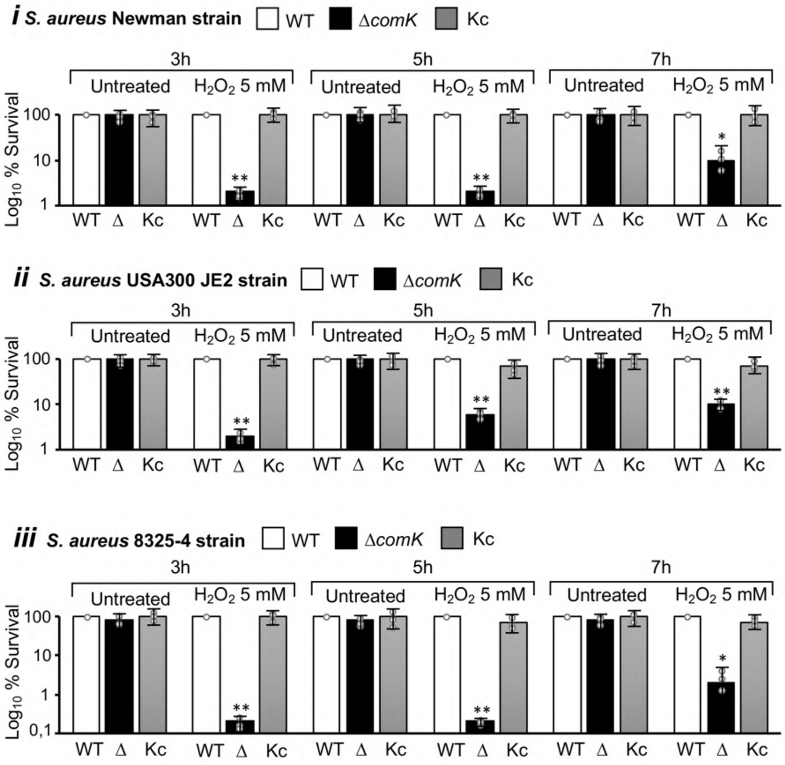

B

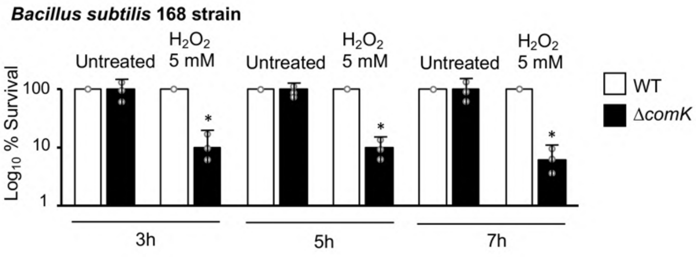

Supplementary Figure 4: The Δ*comK* mutant is more sensitive to oxidative stress in different genetic backgrounds. A) Relative survival rate of different mutants in relation to WT strain in three different staphylococcal genetic backgrounds; Newman (*i*), USA300 JE2 (*ii*) and 8325-4 (*iii*) strains. 10 ml TSB cultures (in 100ml flasks, incubated at 37°C with 200 rpm agitation) were supplemented with H_2O_2 5mM and samples were collected for CFU count at 3, 5 and 7 h upon inoculation. Statistical differences were measured by one-sided ANOVA with Tukey's test for multiple comparison, *$p<0.05$, **$p<0.01$. Data are shown as mean ± SD of three different biological replicates (n = 3). **B)** Δ*comK* mutant in *B. subtilis* (168 strain) showed a growth defect in glucose-based minimal media. Relative percentage of survival of WT and Δ*comK* mutant in MSgg growth media in which glucose was used as carbon source. *comK* in *B. subtilis* is expressed only in glucose-based minimal medium during the transition to stationary phase[5-7]. WT and Δ*comK* mutant were grown in glucose-based defined media to induce natural competence[8,9]. H_2O_2 was added to a final concentration of 5 mM one hour after inoculation. The survival rate was monitored at 3, 5 and 7 h upon inoculation. Significance was measured by two-tailed Student's *t*-test, *$p<0.1$, **$p<0.05$. Data are shown as mean ± SD of three different biological replicates (n = 3). Source data are provided as a Source Data file.

Supplementary Figure 5

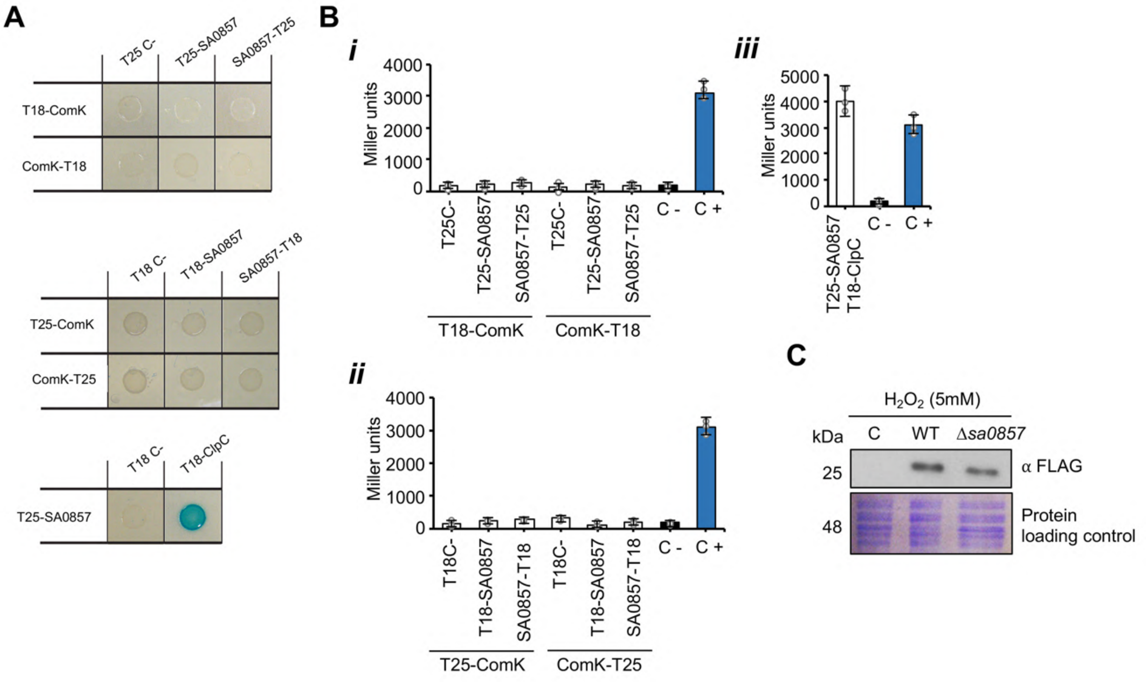

Supplementary Figure 5: MecA does not regulate ComK levels in *Staphylococcus aureus.* **A)** Analysis of protein-protein interactions between ComK and MecA (SA0857) using a bacterial two-hybrid assay (T18 and T25 interaction plasmids from BACTP, EuroMedex)[10]. C- is a control strain harbouring empty plasmids. The SA0857-ClpP interaction[11] was used as a positive control. In *B. subtilis*, MecA represses ComK activity by recruiting ComK to the ClpCP protease for protein degradation[12]. Our bacterial two-hybrid assay showed interaction between SA0857 and ClpP but no interaction between SA0857 and ComK in *S. aureus*. These results are representative of the results obtained in three independent experiments. **B)** Quantitative determination of the ComK-SA0857 interaction using a bacterial two-hybrid analysis. C – is a control strain harboring empty plasmids. C + is a positive control strain, which harbors the plasmids to express the interacting domains of a leucine zipper protein. (i) ComK cloned in T18 plasmid and SA0857 cloned in T25 plasmid. (ii) ComK cloned in T25 plasmid and SA0857 cloned in T18 plasmid. (iii) A positive control that is specific to our assays is represented by a strain that expresses SA0857 and ClpC, as it is known that these two proteins interact[11]. Graphs represented β-galactosidase activity in Miller. Significance was measured by analysis of variance one-sided ANOVA. Data are shown as mean ± SD of three different biological replicates (n = 3). **C)** Immunodetection of ComK levels in diverse ComK-FLAG labelled strains. In the absence of SA0857 (Δ*sa0857* mutant), ComK levels are similar to the WT ones. C is a control unlabelled strain. Protein loading control is shown by the Coomassie stained protein SDS-PAGE. These results are representative of the results obtained in three independent experiments. Source data are provided as a Source Data file.

Supplementary Figure 6

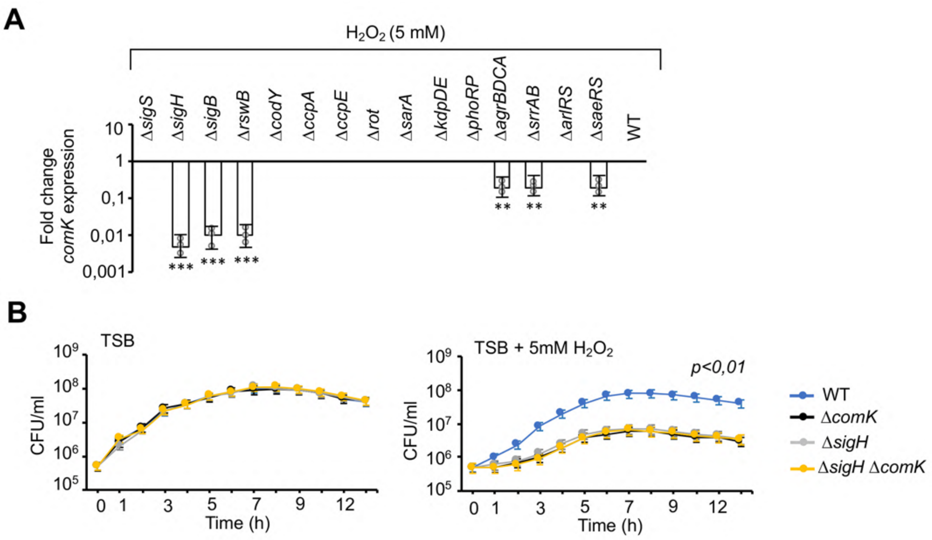

Supplementary Figure 6: *comK* expression in *S. aureus* is induced in response to oxidative stress A) qRT-PCR analysis to quantify *comK* expression in distinct *S. aureus* genetic backgrounds in H_2O_2-treated TBS cultures. *S. aureus* induces the expression of *comK* in the presence of H_2O_2 5 mM. Mutants were grown in these conditions and tested for inhibition of *comK* expression. Statistical differences were measured by one-sided ANOVA with Tukey's test for multiple comparison, ***p*<0.01, ****p*<0.001. Data are shown as mean ± SD of three different biological replicates (n = 3). **B)** Growth curve of WT, Δ*comK*, Δ*sigH* and Δ*sigH* Δ*comK* strains in TSB medium with and without H_2O_2 (5 mM). Cultures were incubated for 12 h at 37°C with 200 rpm agitation. Statistical differences were measured by one-sided ANOVA with Tukey's test for multiple comparison. Data are shown as mean ± SD of three different biological replicates (n = 3). Source data are provided as a Source Data file.

Supplementary Figure 7

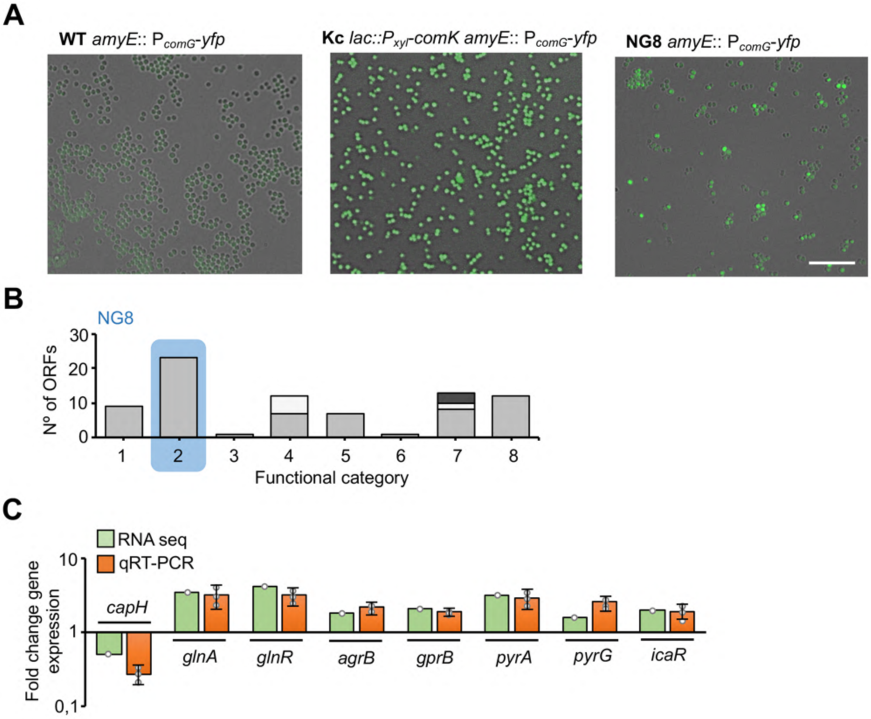

Supplementary Figure 7: The NG8 strain shows higher expression of *comK*. A) Fluorescence microscopy images of *S. aureus* cells from WT, Kc and NG8 strains grown in TSB medium for 24 h at 37°C. These strains harbored the P_{comG}-*yfp* transcriptional fusion integrated in the chromosome to report for ComK activity. The expression of the reporter is not detected in WT cells whereas Kc cells and NG8 cells show high expression of the reporter. Fluorescence signal showed bimodal expression in NG8, pointing to a bifurcation of a subpopulation of cells with higher fluorescence levels. The Kc cells did not show bimodal P_{comG} expression because the Kc strain is genetically engineered to produce ComK under the control of a constitutive promoter. Scale bar is 25 μm. **B)** Classification of ORFs that harbor SNPs in the strain NG8 using TIGRfam, SEED, and Gene Ontology functional categories. For each category, columns represent the number of regulated genes. Group 1 - protein metabolism; group 2 - carbohydrate metabolism and respiration; group 3 - amino acid metabolism; group 4 - stress response genes; group 5 - virulence; group 6 - iron acquisition; group 7 - DNA metabolism (gray), cell division (white) and cell envelope (dark green); group 8 - other genes. Group 2 genes showed higher representation in NG8 strain (highlighted in blue). **C)** qRT-PCR analysis of benchmark genes to validate RNA-Seq analysis. Data are shown as mean ± SD of three different biological replicates (n = 3). Expression of genes were compared to transcriptomic data. Data shows fold-change gene expression of Δ*comK* mutant in relation to WT strain. Significance was measured by two-tailed Student's *t* test and no significant differences were detected. Source data are provided as a Source Data file.

Supplementary Figure 8

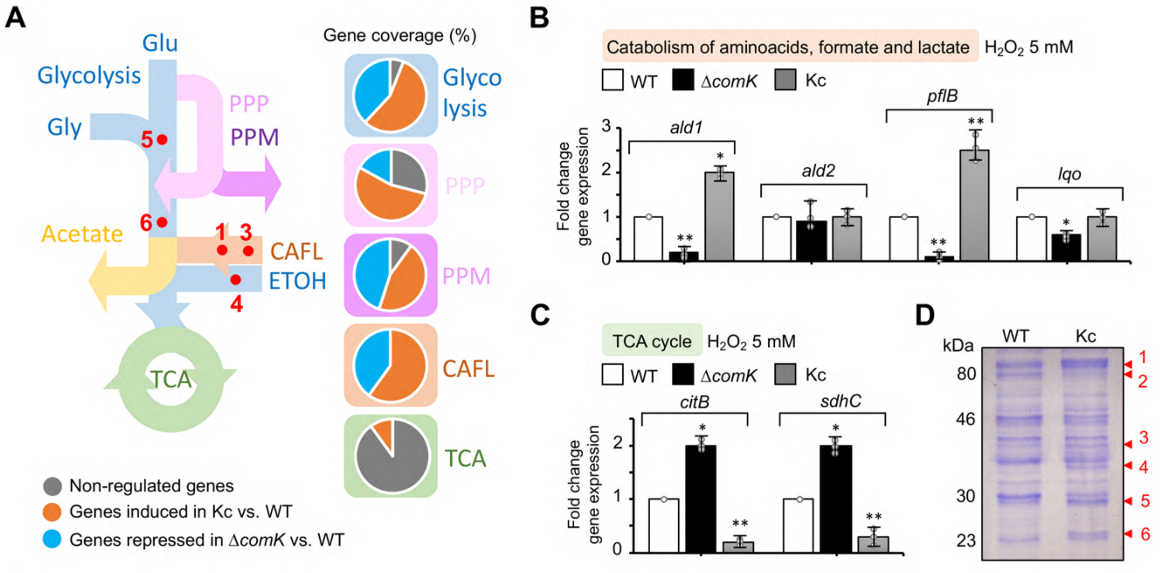

E

1 Formate acetyltransferase (79%)
D0K7Y0_STAAD (85 kDa)
GLYCOLYSIS

2 ATP-dependent Clp protease ClpC (75%)
A6QK38_STAAE (91 kDa)
PROTEIN METABOLISM

3 Alanine dehydrogenase 1 (61%)
DHA1_STAA8 (40.2 kDa)
GLYCOLYSIS

4 Alcohol dehydrogenase (63%)
Q2YSX0|ADH_STAAB (36 kDa)
FERMENTATION

5 Fructose-bisphosphate aldolase (88%)
ALF1_STAA3 (33 kDa)
GLYCOLYSIS

6 2,3-bisphosphoglycerate-dependent phosphoglycerate mutase (85%)
D0K8Y9|D0K8Y9_STAAD (26.7 KDa)
GLYCOLYSIS

Supplementary Figure 8: ComK enhances the glycolytic flux to feed the fermentative metabolism of *S. aureus*. A) Left panel; *S. aureus* glucose catabolism pathway. Glucose metabolism (Glu, blue arrow), glycerol catabolism (Gly, blue arrow), pentose phosphate pathway (PPP, pale pink arrow), purines and pirimidines biosynthesis pathway (PPM, dark pink arrow), catabolism of amino acids, formate and lactate (CAFL, brown arrow), ethanol metabolism (blue arrow), acetate metabolism (yellow arrow) and tricarboxylic acid cycle (TCA, green arrow). Right panel; pie charts of gene coverage (%) for each catabolic pathway of genes induced in Kc vs. WT (orange) or repressed genes in Δ*comK* vs. WT (blue). **B)** Expression of genes involved in pyruvate production via catabolism of amino acids, formate or lactate. Alanine is converted to pyruvate by alanine dehydrogenase 1 or 2 (*ald1* or *ald2*). Formate is transformed to pyruvate by formate acetyltransferase (*pflB*). Lactate is converted to pyruvate by lactate-quinone oxidorreductase (*lqo*). qRT-PCR analysis of *ald1-2*, *pflB* and *lqo* in different *S. aureus* strains growing in TSB medium treated with H_2O_2 5 mM. The Δ*comK* mutant showed a reduction in the expression of the genes whereas the Kc strain showed an induction of gene expression. This suggests that *comK* expression is linked to the activation of the catabolic pathways to pyruvate production. Significance was measured by analysis of variance one-sided ANOVA with Tukey's test for multiple comparison, *p<0.05, **p<0.01. Data are shown as mean ± SD of three different biological replicates (n = 3). **C)** Expression of genes involved in TCA cycle. The isomerization of citrate to isocitrate is catabolized by an aconitate hydratase (*citB*). Succinate is transformed to fumarate by succinate dehydrogenase (*sdhCBA*). qRT-PCR analysis of *citB* and *sdhC* in different *S. aureus* strains growing in TSB medium treated with H_2O_2 5 mM. The Δ*comK* mutant showed an induction of *citB* and *sdhC* expression whereas the Kc strain showed repression of these genes. This result suggests that *comK* repression is associated with an induction of the TCA cycle. Significance was measured by analysis of variance one-sided ANOVA with Tukey's test for multiple comparison, *p<0.05, **p<0.01. Data are shown as mean ± SD of three different biological replicates (n = 3) **D)** SDS-PAGE showing the protein profile of WT and Kc strains grown in TSB 37°C, 200 rpm. The protein profiles of WT and Kc cell extracts showed differences in specific protein bands. In particular, the ones highlighted in 1, 3, 4, 5 and 6 show increased abundance in the Kc protein profile. An extra

band was also detected in position 2 of the WT protein profile. These results are representative of the results obtained in three independent experiments. **E)** Identification of the proteins from 1, 3, 4, 5 and 6 bands that are increased in Kc strain and the percentage of identity coverage obtained by mass spectrometry analysis. They are glycolytic enzymes and they catalyse the reactions highlighted with red dots in the scheme of panel A. Source data are provided as a Source Data file.

Supplementary Figure 9

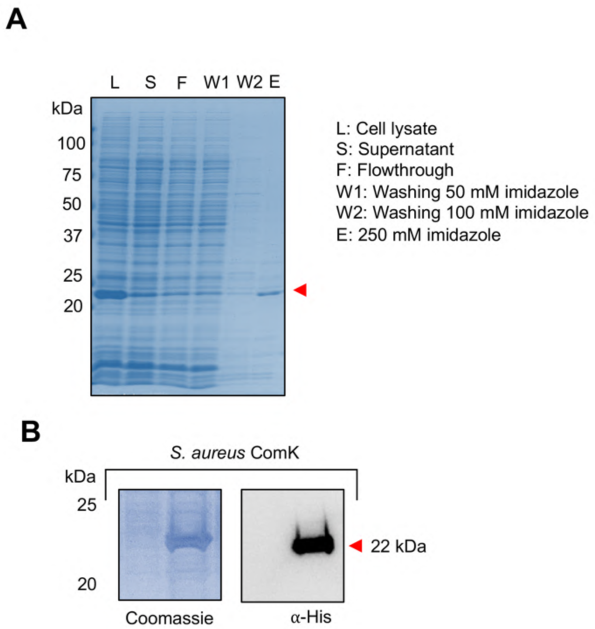

Supplementary Figure 9: Overproduction and purification of ComK. A) SDS-PAGE that resolves the samples from ComK purification steps. The band attributable to ComK is marked with a red arrowhead. A His6-tagged version of ComK was overproduced in *E. coli* and purified using a Ni-binding resin. **B)** Immunodetection of ComK in samples from ComK purification steps using monoclonal antibodies against His6 tag (Rockland). These results are representative of the results obtained in three independent experiments. Source data are provided as a Source Data file.

Supplementary Figure 10

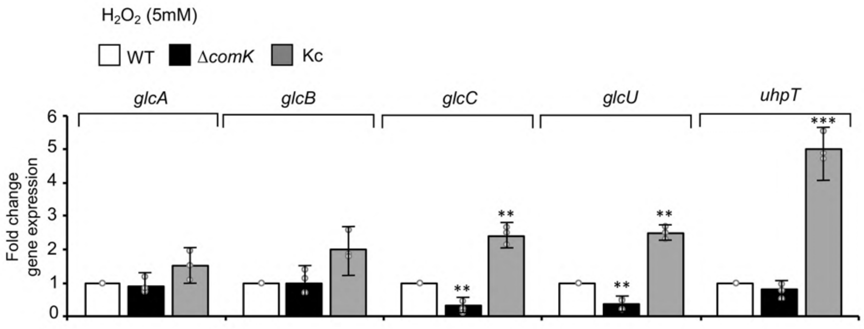

Supplementary Figure 10: The Δ*comK* mutant showed lower *glcC, glcU* and *uhpT* expression than WT. qRT-PCR analysis of *glcA*, *glcB*, *glcC*, *glcU* and *uhpT* genes in different *S. aureus* strains in H_2O_2-treated TSB cultures. Statistical differences were measured by one-sided ANOVA with Tukey's test for multiple comparison ***p*<0.01, ****p*<0.001. Data are shown as mean ± SD of three different biological replicates (n = 3). Source data are provided as a Source Data file.

Supplementary Figure 11

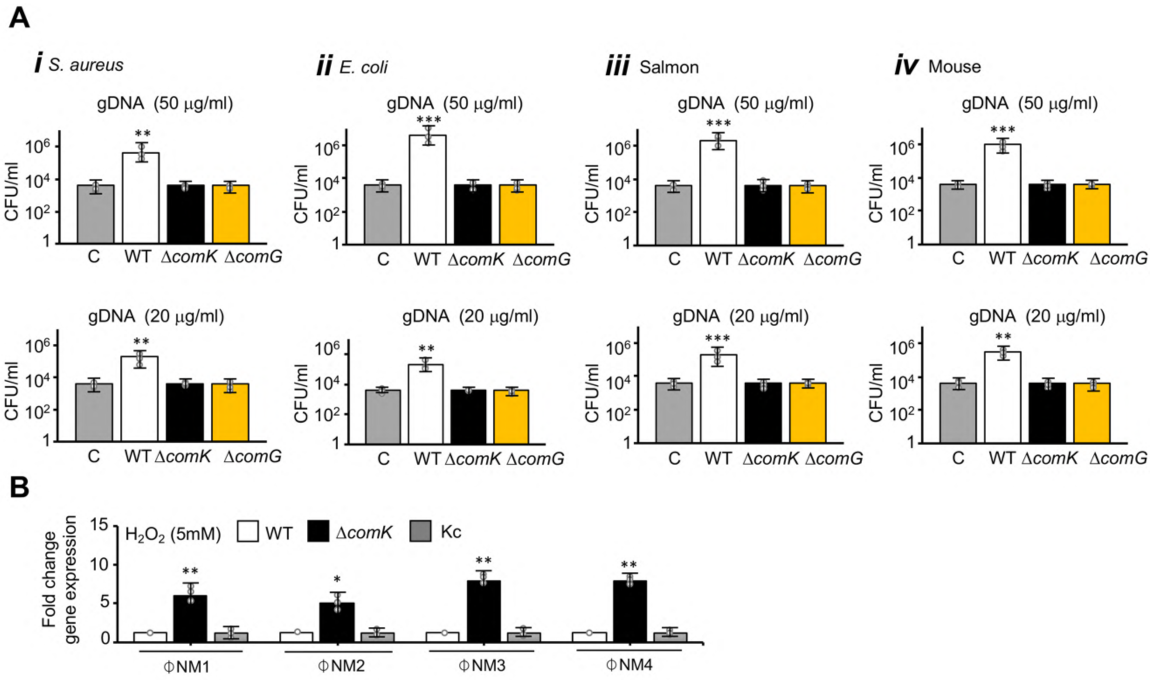

Supplementary Figure 11: Growth of *S. aureus* using DNA as nutrient source. A) Determination of growth yields (CFU/ml) in SMM cultures (37°C, overnight incubation, 200 rpm agitation) supplemented with DNA as nutrient source. DNA was isolated from different sources (*i S. aureus*, *ii E. coli*, *iii* salmon sperm and *iv* mouse tail) at 20 or 50 µg/ml. Control sample (C) represents cultures with no DNA supplement. Statistical differences were measured by one-sided ANOVA with Tukey's test for multiple comparison; $^{**}p<0.01$, $^{***}p<0.001$. Data are shown as mean ± SD of three different biological replicates (n = 3). **B)** qRT-PCR analysis to quantify induction of prophage expression in H_2O_2-treated TSB cultures of different *S. aureus* strains. *S. aureus* Newman strain (WT) contains 4 prophages (ϕNM1-4) in its chromosome[13]. Differences were examined by one-sided ANOVA with Tukey's test for multiple comparison; $^{*}p<0.05$, $^{**}p<0.01$. Data are shown as mean ± SD of three different biological replicates (n = 3). Source data are provided as a Source Data file.

Supplementary Figure 12

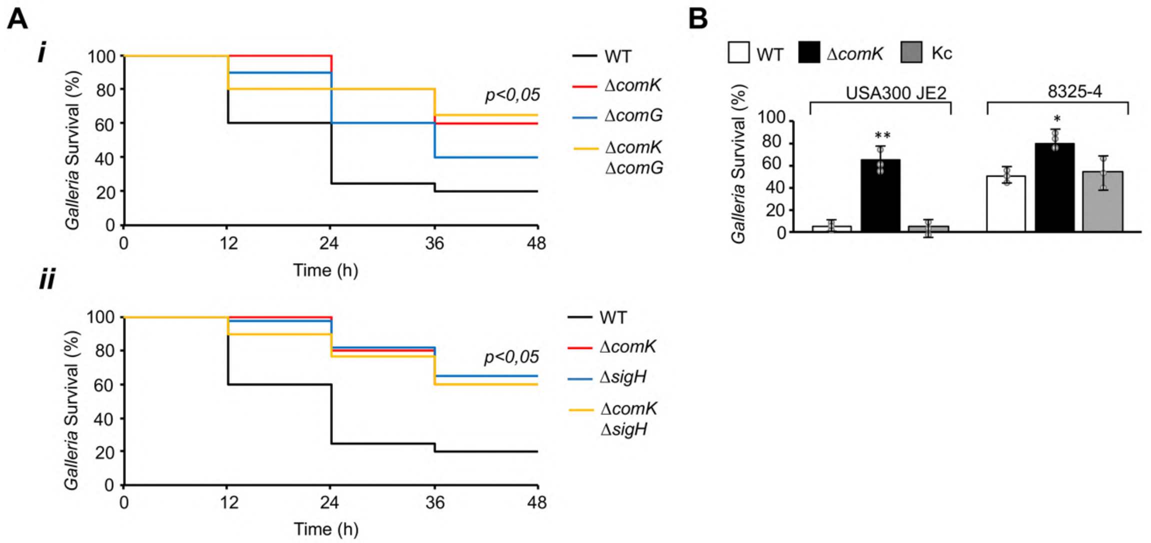

Supplementary Figure 12: *comK* expression is required for infection in different *S. aureus* strains. A) *In vivo* infections of different mutants using the invertebrate model *Galleria mellonella*. In panel *i*, *Galleria mellonella* were infected with WT, Δ*comK*, Δ*comG* and Δ*comK* Δ*comG* strains (10^6 CFU). Surviving larvae were counted after 48 h of incubation (n = 15 larvae/group; 3 independent experiments). In panel *ii, Galleria mellonella* were infected with WT, Δ*comK*, Δ*sigH* and Δ*comK* Δ*sigH* strains (10^6 CFU). Statistical significance was measured by ANOVA with Tukey's test for multiple comparison (n = 15). **B)** *In vivo* infections of the different mutants in distinct *S. aureus* genetic backgrounds; USA300 JE2 and 8325-4 strains (10^6 CFU), using the invertebrate model *Galleria mellonella*. Surviving larvae were counted after 48 h of incubation (n = 15 larvae/group; 3 independent experiments). One-sided ANOVA with Tukey's test for multiple comparison; **p*<0.05, ***p*<0.01. Data are shown as mean ± SD of three different biological replicates (n = 3). Source data are provided as a Source Data file.

Supplementary Figure 13

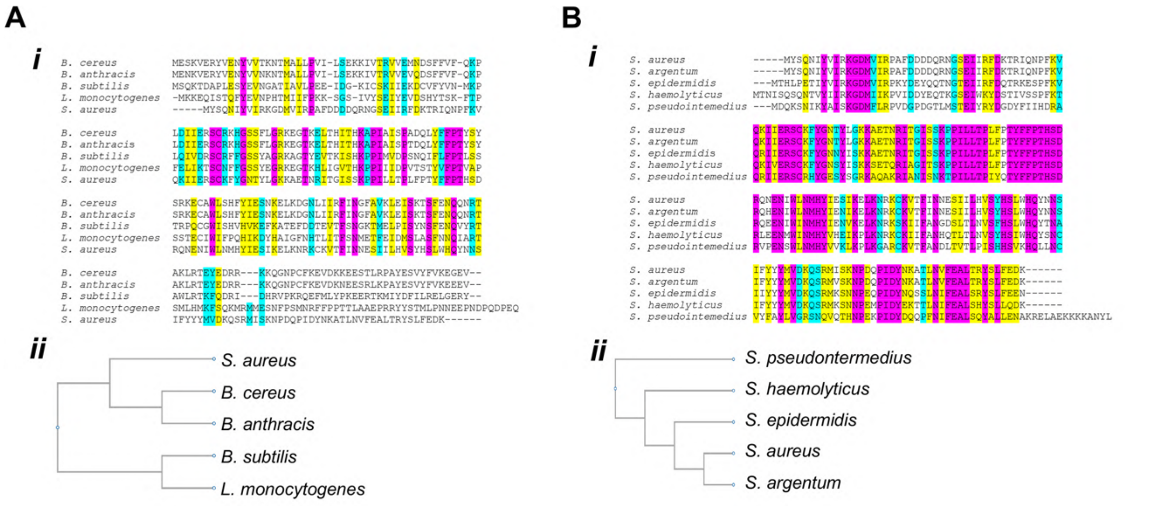

Supplementary Figure 13: Conservation of ComK in firmicutes A) (*i*) Multiple alignment of ComK homologues from *Staphylococcus aureus* and closely-related species. Conserved amino acids are detected throughout the entire protein although they concentrate in the central region. The most different amino acid sequences were detected at the N- and C-terminal parts. Aminoacids highlighted in purple, yellow and blue indicate identical residues in all sequences, conserved substitutions and semi-conserved substitutions, respectively. Alignments were made using CLUSTALW (www.ebi.ac.uk/clustalw)[14]. (*ii*) Phylogenetic relationship of ComK from *Staphylococcus aureus* and closely-related species. The phylogenetic distance is represented according to the % of amino acid identity between two different sequences. **B) (***i*) Multiple alignment of ComK homologues from distinct *Staphylococcal species*. Aminoacids are conserved throughout the entire protein. The differences in the amino acid sequence concentrate at the N-terminal part. (*ii*) Phylogenetic relationship of ComK from distinct Staphylococcal species.

Supplementary Tables

Supplementary Table 1: Growth conditions that induce *comK* expression and DNA uptake in *S. aureus*. WT is wild-type strain whereas Kc is a strain that expresses *comK* constitutively. DNA uptake efficiency is measured as the number of *erm*$^+$ *lac*$^+$ CFU in relation to the total CFU count. DNA uptake experiments were performed using plasmid DNA as well as chromosomal DNA, following the protocol reported by Morikawa *et al*[15]. Extracellular DNA harboring *erm*$^+$ and *lacZ*$^+$ markers was used to identify positive colonies. Plasmid DNA (pMAD plasmid) or genomic DNA was used for experiments at a final concentration of 2 μg/ml. Positive colonies were detected using plasmid DNA.

Condition tested TSB medium	Strain	Fold-change *comK* expression	*erm*$^+$ *lac*$^+$ CFU/ Total CFU
Untreated	WT	1.0	n.d.
	Kc	12	$6 \times 10^{-9} \pm 3 \times 10^{-10}$
H_2O_2 5 mM	WT	9	$6 \times 10^{-9} \pm 1 \times 10^{-9}$
	Kc	12	$6 \times 10^{-9} \pm 1 \times 10^{-10}$
pH = 4	WT	10	$3 \times 10^{-9} \pm 1 \times 10^{-9}$
	Kc	12	$2 \times 10^{-9} \pm 1 \times 10^{-10}$
Mupirocin 1 mM	WT	9	$1 \times 10^{-8} \pm 6 \times 10^{-9}$
	Kc	12	$2 \times 10^{-9} \pm 5 \times 10^{-10}$
45°C incubation	WT	1.0	n.d.
pH = 9	WT	1.0	n.d.
Erythromycin 1 mM	WT	1.0	n.d.
Oxacillin 2 mM	WT	1.0	n.d.
Vancomycin 1 mM	WT	1.0	n.d.
Plate desiccation	WT	1.0	n.d.
EDTA 1mM	WT	1.0	n.d.

Supplementary Table 2: Identification of SNPs in the genome of the strain NG8 using whole genome sequencing. A total of 114 SNPs were detected. 2 SNPs caused nonsense mutations (Table highlighted in orange) and 48 SNPs cause missense mutations with moderate impact in the encoded amino acid sequence (Table highlighted in dark blue). This is a substitution to an aminoacid with different physicochemical properties. 11 SNPs caused missense mutations with low impact. This is a substitution to an amino acid with similar physicochemical properties (Table highlighted in pale blue). 18 SNPs caused silent mutations. This is a mutation in the ORFs that does not cause an amino acid substitution (Table highlighted in pale orange). 35 SNPs were located in intergenic regions (data not shown).

Nonsense mutations					
**Gene**	**Name**	**Description**	**TIGRFAM role**	**Genome position**	**Amino acid substitution**
NWMN_1947	*pfkB*	Carbohydrate kinase	Glucolysis. Metabolism of sugars	308619	W-Stop
NWMN_0922	*atl*	Autolysin precursor	Cell envelope	1024531	Y-Stop

Missense mutations (Moderate impact)					
**Gene**	**Name**	**Description**	**TIGRFAM role**	**Genome position**	**Amino acid substitution**
NWMN_0049	*nptA*	Na/Pi transporter	Transport of cations	65889	G-S
NWMN_0109	*capO*	Capsular polysaccharide biosynthesis	Pentose phosphate pathway. Carbohydrate metabolism	1347274	E-K
NWMN_0177	-	Ribonucleoside hydrolase	Glycosylase. Carbohydrate metabolism	231682	D-N
NWMN_0188	*tarF*	Glycerol-phosphate transferase	Teichoic acid biosynthesis. Cell envelope	243910	P-S
NWMN_0244	-	Unknown	Unknown	298649	S-N
NWMN_0261	-	Unknown	Unknown	322346	T-I
NWMN_0294	-	Capsid protein	Phages	343558	M-I
NWMN_0398	*hsdM1*	Restriction system	Virulence and defense	461098	D-N
NWMN_0467	-	Putative glucose transporter PTS family	Carbohydrate metabolism and respiration	531922	P-S
NWMN_0486	*mcsB*	Arginine kinase	Stress response	558195	S-F
NWMN_557	-	Nucleotide disulfide oxidoreductase	Energy metabolism. Respiration	641555	T-A
NWMN_0574	-	Acetyltransferase	Synthesis of cofactors. Metabolism of proteins	655170	D-N
NWMN_0596	*mnhD*	Cation/H+ antiporter	Energy metabolism. Respiration	676945	G-E
NWMN_0606	*tagA*	N-acetyl mannosaminyl transferase	Glycosyltransferase. Cell envelope. Carbohydrate metabolism	686713	G-D
NWMN_0790	-	unknown	Unknown	880538	G-D
NWMN_0846	-	LysR transcriptional regulator	Gene regulation	942473	G-D
NWMN_0935	*purC*	Phosphoribosyl carboxamide synthase	Nucleotide biosynthesis. DNA metabolism	1038224	G-E
NWMN_0938	*purF*	Amidophosphoribosyl transferase	Nucleotide biosynthesis. DNA metabolism	1041601	G-F

NWMN_0952	*cydA*	Cytochrome ubiquinol oxidase subunit I	Electron transport. Respiration	1058694	G-D
NWMN_0953	*cydB*	Cytochrome D ubiquinol oxidase subunit II	Electron transport. Respiration	1058864	G-D
NWMN_0979	*pycA*	Pyruvate carboxylase	Glycolysis. Metabolism of sugars	1085703	S-N
NWMN_1174	*polC*	DNA polymerase III subunit alpha	Cell division	1292880	D-N
NWMN_1209	*glpD*	Glycerol-3-phosphate dehydrogenase	Glycolysis. Metabolism of sugars	1335006	S-N
NWMN_1236	*nuc2*	Thermonuclease	Virulence and defense	1359654	E-K
NWMN_1258	*sbcC*	Exonuclease	Virulence and defense	1379466	E-K
NWMN_1293	*opp*	Peptide ABC transporter	Transport	1424346	S-F
NWMN_1452	*glpG*	Intramembrane serine protease	Stress response	1620299	R-H
NWMN_1764	-	Unknown	Unknown	1976644	D-N
NWMN_1769	-	Phage amidase	Phages	1981481	E-K
NWMN_1813	-	Thioesterase	Carbohydrate metabolism and respiration	2021830	T-I
NWMN_1886	-	Phage minor structural protein	Phages	2099605	D-N
NWMN_1985	*cshA*	RNA helicase	Stress response	2207121	A-T
NWMN_2091	-	NADP-dependent oxidoreductase	Energy metabolism. Respiration	2328801	G-E
NWMN_2459	*ldhD*	Lactate dehydrogenase	Energy metabolism. Respiration	2706441	G-D
NWMN_2543	-	Glucosaminidase	Carbohydrate metabolism and respiration	2803587	A-T
NWMN_1665	*arsB*	Arsenical efflux pump membrane protein ArsB	Membrane transport	1862843	G-D
NWMN_1230	*cls1*	Cardiolipin synthase	Energy metabolism. Respiration	2213656	R-C
NWMN_1482	*dnaJ*	Chaperone protein	Stress response	1648142	E-K
NWMN_1470	*era*	GTP-binding protein	Protein metabolism	1638843	D-N
NWMN_0223	*essC*	T7SS protein	Virulence and defense	281470	G-S
NWMN_0509	*fusA*	Elongation factor G	Protein synthesis. Protein metabolism	586929	H-N
NWMN_0436	*gltB*	Glutamate synthase	Nitrogen metabolism. Respiration	487387	D-N
NWMN_0692	*hisC*	Histidinol-phosphate aminotransferase	Amino acid biosynthesis	776832	E-K
NWMN_0403	*lpl10*	Tandem lipoprotein	Virulence and defense	1235751	D-N
NWMN_1641	*rsmB*	tRNA-methyltransferase	Protein synthesis. Protein metabolism	1235751	R-Q
NWMN_0524	*sdrD*	Staphylococcal Adhesin	Virulence and defense	608514	D-N
NWMN_1162	*xerC*	Recombinase	DNA metabolism	1277195	D-N

Missense mutations (Low impact)					
**Gene**	**Name**	**Description**	**TIGRFAM role**	**Genome position**	**Amino acid substitution**
NWMN_0179	*ptsN*	Nitrogen-regulatory protein	Metabolism of carbohydrates and respiration	234676	A-V
NWMN_0468	*mazG*	Nucleotide pyrophosphohydrolase	DNA metabolism	532975	A-V
NWMN_1856	*pncA*	Cysteine hydrolase	DNA metabolism	2069806	A-V
NWMN_1904	-	dUTP pyrophosphatase	DNA metabolism	2116663	V-I
NWMN_1939	-	Membrane metalloprotease	Stress response	2147093	A-V
NWMN_1978	-	Unknown	Unknown	2195713	V-I
NWMN_1936	*mtlA*	Mannitol phosphotransferase	Metabolism of carbohydrates and respiration	2281016	A-V
NWMN_2328		ABC transporter	Membrane transport	2559771	A-V
NWMN_1578	*dnaB*	Replication initiation protein	DNA metabolism	21500	A-V
NWMN_0796	*lipA*	Lipoyl synthase	Biosynthesis of cofactors. Protein metabolism	886759	A-V
NWMN_0726	*uvrB*	Excinuclease ABC subunit B	DNA metabolism	814759	A-I

Silent mutations					
**Gene**	**Name**	**Description**	**TIGRFAM role**	**Genome position**	**Amino acid substitution**
NWMN_0320	*lplA2*	Lipoate ligase A	Metabolism of cofactors. Protein metabolism	369888	Silent (Q)
NWMN_0338	*fepC*	Iron permease	Metabolism of iron	388675	Silent (G)
NWMN_0419	*mpsB*	Na/H^+ transporter	Energy metabolism. Respiration	469505	Silent (R)
NWMN_421	-	Phosphatase	Unknown	473153	Silent (A)
NWMN_593	*mnhA2*	Na/H^+ antiporter	Energy metabolism. Respiration	674446	Silent (L)
NWMN_0925	-	Teichoic acid transferase	Cell envelope	1026937	Silent (H)
NWMN_1256	-	Unknown	Unknown	1379330	Silent (G)
NWMN_1754		2-hydroxyacid dehydrogenase	Metabolism of carbohydrates and respiration	1965338	Silent (S)
NWMN_1888	-	Phage tail protein	Phages	2103883	Silent (L)
NWMN_2444	-	Thiol-disulphide oxidoreductase	Protein synthesis. Protein metabolism	2261565	Silent (A)
NWMN_2542	-	Phage infection protein	Phages	2800650	Silent (R)
NWMN_1431	*accC*	Acetyl-CoA carboxylase	Metabolism of carbohydrates and respiration	1603381	Silent (G)
NWMN_1519	*alaS*	Alanyl-tRNA synthetase	Protein synthesis. Protein metabolism	1683219	Silent (Y)
NWMN_1145	*ftsY*	Docking protein	Cell division	1256795	Silent (E)

NWMN_2567	*icaB*	Adhesion protein B	Biofilm formation. Pathogenesis and defense	2835068	Silent (I)
NWMN_1355	*msrA*	Methionine sulfoxide reductase	Protein modification and repair. Stress response	1468854	Silent (K)
NWMN_1194	*recA*	Recombinase A	DNA repair. Stress response	1316958	Silent (D)
NWMN_0722	*secA*	Protein translocase	Protein fate. Protein metabolism	807982	Silent (I)

Supplementary Table 3. RNA-Seq dataset. Read analysis statistics.

**Libraries**	**Δ**	**W**	**Kc**	**W**
Number of input reads	12398206	8777842	12168481	10292257
N°. of reads - PolyA detected and removed	2368924	1171777	1495392	1259427
N°. of reads - Single 3' A removed	2059780	1797308	2475611	2066656
N°. of reads - Unmodified	7969502	5808757	8197478	6966174
N°. of reads - Removed as too short	419436	49123	68887	71015
N°. of reads - Long enough and used for alignment	11978770	8728719	12099594	10221242
Total N°. of aligned reads	8879512	8057305	10986593	9798324
Total N°. of unaligned reads	3099258	671414	1113001	422918
Total N°. of uniquely aligned reads	8496603	7736035	10798577	9604763
Total N°. of alignments	9441384	8506611	11282881	10073274
Total N°. of split alignments	0	0	0	0
% of aligned reads (to N°. of input reads)	71,62	91,79	90,29	95,2
% of aligned reads (to N°. of long enough reads)	74,13	92,31	90,80	95,86
% of uniquely aligned reads (to aligned reads)	95,69	96,01	98,29	98,02
N°. of aligned reads	8879512	8057305	10986593	9798324
N°. of uniquely aligned reads	8496603	7736035	10798577	9604763
N°. of alignments	9441384	8506611	11282881	10073274
N°. of split alignments	0	0	0	0

Supplementary Table 4: List of strains used in this work

**Strain**	**Description**	**Source**
S. aureus		
S. aureus Newman	WT	[16]
S. aureus Newman	Δ*comK::km* (Δ strain)	This study
S. aureus Newman	Δ*comK::km, amyE:* P_{xyl}*-comK* (Kc strain)	This study
S. aureus Newman	Δ*comG*::*km*	This study
S. aureus Newman	Δ*sae::km*	[3]
S. aureus Newman	Δ*srrA::tet*	[17]
S. aureus Newman	Δ*agr::tet*	[3]
S. aureus Newman	Δ*kdp::tet*	[17]
S. aureus Newman	Δ*arl*::*mls*	[18]
S. aureus Newman	Δ*rswB*::*mls*	[19]
S. aureus Newman	Δ*rot*::*tet*	[20]
S. aureus Newman	Δ*sarA*::*tet*	[21]
S. aureus Newman	Δ*codY*::*tet*	[22]
S. aureus Newman	Δ*sigB*::*mls*	[3]
S. aureus Newman	Δ*sigH::mls*	This study
S. aureus Newman	Δ*sigS*::*mls*	This study
S. aureus Newman	Δ*sa0857::km*	This study
S. aureus Newman	Δ*comG::km*	This study
S. aureus Newman	Ω*comG::mls*	This study
S. aureus Newman	Δ*sigH::mls* Δ*comK::km*	This study
S. aureus Newman	Ω*comG::mls* Δ*comK::km*	This study
S. aureus Newman	WT *amyE*::P_{comG}-*yfp*	This study
S. aureus Newman	NG08 strain *amyE*::P_{comG}-*yfp*	This study
S. aureus Newman	lacA::P_{xyl}-*comK, amyE*::P_{comG}-*yfp*	This study
S. aureus 8325-4	WT	[23]
S. aureus 8325-4	Δ*comK::km* (Δ strain)	This study
S. aureus 8325-4	Δ*comK::km, amyE:* P_{xyl}*-comK* (Kc strain)	This study
S. aureus USA300 JE2	WT	[24]
S. aureus USA300 JE2	Δ*comK::km* (Δ strain)	This study
S. aureus USA300 JE2	Δ*comK::km, amyE:* P_{xyl}*-comK* (Kc strain)	This study
S. aureus USA300 JE2	Ω*sigS::mls*	[24]
S. aureus USA300 JE2	Ω*sigH::mls*	[24]
S. aureus USA300 JE2	Ω*sigB*::*mls*	[24]

S. aureus USA300 JE2	Ω*rswB*::*mls*	[24]
S. aureus USA300 JE2	Ω*codY*::*mls*	[24]
S. aureus USA300 JE2	Ω*rot*::*mls*	[24]
S. aureus USA300 JE2	Ω*sarA*::*mls*	[24]
S. aureus USA300 JE2	Ω*kdp*::*mls*	[24]
S. aureus USA300 JE2	Ω*pho*::*mls*	[24]
S. aureus USA300 JE2	Ω*agr*::*mls*	[24]
S. aureus USA300 JE2	Ω*arl*::*mls*	[24]
S. aureus USA300 JE2	Ω*kat*::*mls*	[24]
S. aureus Newman	Ω*glcC*::*mls*	[24]
S. aureus RN4220	*rbsU*$^-$, *tcaA*$^-$, ΔΦ11, ΔΦ12, ΔΦ13, r- m-	[25]
S. aureus SCV1	*Clinical isolate hemD*$^-$	[26]
S. aureus SCV2	*Clinical isolate hemD*$^-$	[26]
S. aureus 8325-4	*ΔhemB*	[27]
B. subtilis		
168	WT	[28]
168	*amyE::*P_{comG}*-yfp*	[29]
168	Δ*comK::spc*	[5,30]
E. coliΩ		
BTH101	pUT18-*comK*, pKT25	This study
BTH101	pUT18C-*comK,* pKT25	This study
BTH101	pUT18-*comK*, pKNT25-*mecA*	This study
BTH101	pUT18-*comK*, pKNT25C-*mecA*	This study
BTH101	pUT18C-*comK*, pKNT25-*mecA*	This study
BTH101	pUT18C-*comK*, pKNT25C-*mecA*	This study
BTH101	pUT18, pKNT25-*comK*	This study
BTH101	pUT18-*mecA* pKT25-*comK*	This study
BTH101	pUT18C-*mecA* pKT25-*comK*	This study
BTH101	pUT18, pKNT25C-*comK*	This study
BTH101	pUT18-*mecA* pKT25C-*comK*	This study
BTH101	pUT18C-*mecA* pKT25C-*comK*	This study
DH5α	pET28a *comK*-His6	This study
BL21 Gold	pET28a *comK*-His6	This study

Supplementary Table 5: List of primers used in this work

Primer	Sequence 5'-3'	Use
**Primers for genetic manipulation**		
AYG-37	tttttgtcgacggtttaaaacttaataaagcgagg	Δ*comK*
AYG-38	tttttgctagcattaagttaatttgcaatagatacc	Δ*comK*
AYG-107	Cctatacacctcaaatggttcgctagaaaacctcgctttattaagtt	Δ*comK*::*km*
AYG-109	gagcgcctacgaggaatttgtatcgatatttgtgcttgatattcaa	Δ*comK*::*km*
AYG-106	gagaacaacctgcaccattgcaagaaaacctcgctttattaagtt	Δ*comK*::*tet*
AYG-108	gggatcaactttgggagagagttcatatttgtgcttgatattcaa	Δ*comK*::*tet*
AYG-124	atgggaattcgaatcaatgtatttc	Kc
AYG-125	ctttttaagcttaagcaaaacctcgctttattaag	Kc
AYG-152	gagcgcctacgaggaatttgtatcgcgatattaattaagcaacgc	Δ*sa0857*
AYG-153	tttttccatggccatgataatcacaaggcgcctc	Δ*sa0857*
AYG-154	cctatcacctcaaatggttcgctgtcatctcactccttacagtcg	Δ*sa0857*
AYG-155	tttttggatccttgcacatcttgccgtaaagc	Δ*sa0857*
AYG-143	tttttgcatccttagaaaatagtggtgaaaacc	Δ*comG*
AYG-144	cctatcacctcaaatggttcgctgcacctcctacatataatcacg	Δ*comG*
AYG-145	cctatcacctcaaatggttcgctgcacctcctacatataatcacg	Δ*comG*::*km*
AYG-146	gagcgcctacgaggaatttgtatcgcttactagtatttaaatgag	Δ*comG*::*km*
AYG-156	gcttgaaacccaggacaataacc	*km*
AYG-157	actccgcatacagcacgataatc	*km*
AYG-158	cctcattattggagggtgaaatg	*tet*
AYG-159	gataggacccaatgaatataatgg	*tet*
AYG-260	ctcggagccacctgctgatcctctaccaccaaagcctttatattc	FLAG tag
AYG-261	ggatcagcaggtggctccgagttcgactacaaagaccatgacgg	FLAG tag
AYG-262	gttcacaagggtttgaatcattactatttatcgtcgtcatctttg	FLAG tag
AYG-263	gaatataaaggctttggtggtagaggatcagcaggtggctccgag	FLAG tag
B2HcomKfw	ttttggatccatccagctaagattaatc	B2H
B2HcomKrv	ttttgtcgacccatgtctctcctttgcgtatc	B2H
B2HmecAfw	ttttggatccatttcttaaaaatgtaagatgtgttag	B2H
B2HmecArv	ttttgccatggcataaatcaacttgtaattgccac	B2H
Pet28comKfw	atataccatgggcatgtattctcaaaatatttatgtgatacgc	ComK purification
Pet28comKrv	ggtgctcgagtttatcttcaaataaagaatagcgtgtc	ComK purification
PcomGAfw	ccgcaattgaaaaaacacctcctacatataatcacg	*comGA* promoter
PcomGArv	ccgcaattgatatcggagcagtcgatgatatag	*comGA* promoter

PglcAfw	ccggaattccttgtccaacctccaaagttg	*glcA* promoter
PglcArv	ccggaattcctacaatattaatcagttaaagcctgttca	*glcA* promoter
PglcBfw	ccggaattcctataaacatcccctatctcaatttccg	*glcB* promoter
PglcBrv	ccggaattcctcgaagaggcattagcacaa	*glcB* promoter
PuhpTfw	ccggaattccaagttttaataaccataaataacatgatataagcaaa	*uhpT* promoter
PuhpTrv	ccggaattcctctctgtcacctcaatcattttcg	*uhpT* promoter
PackAfw	ccggaattcctaagtaatgatcctccatcgtttg	*ackA* promoter
PackArv	ccggaattcctaggctattatccaattgatgaaagaa	*ackA* promoter
PglcCfw	ccggaattcctcaacatccctcctaattattctcaat	*glcC* promoter
PglcCrv	ccggaattcctattcttgtcaactgattgattacctg	*glcC* promoter
PglcUfw	ccggaattccatttgtttttatttgttagcgaaggttt	*glcU* promoter
PglcUrv	ccggaattccagactactcactctcctatactatttatg	*glcU* promoter
PcidCfw	ccggaattccttattactaatagcctccctttctgtc	*cidC* promoter
PcidCrv	ccggaattccttataaaaatagagagaaaattaaagacaatgtctc	*cidC* promoter
Ppgmfw	ccggaattccggcgttaaatacgttgttatcgg	*pgm* promoter
Ppgmrv	ccggaattcctgattattttgcaccttctaacaattgt	*pgm* promoter
PpfkAfw	ccggaattcctgacgacatacctttctataactgattt	*pfkA* promoter
PpfkArv	ccggaattccaattgcagctgaaacaatgaaaattac	*pfkA* promoter
RW327	atgacaatgccgattataatg	SIP-DNA
RW330	caaagcaggtcttactacagg	SIP-DNA
**Primers for RT-PCR analyses**		
comKF	agagacaaaccgcattactg	*comK*
comKR	gccataaactgtggtatgaaac	*comK*
AYG-249	gcggtaatacgtaggtggcag	16S rna
AYG-250	tttccaatgaccctccacgg	16S rna
ackAF	gggtattcgtgctttccgta	*ackA*
ackAR	tcaggcattgtttgatggaa	*ackA*
cidCF	gtacaatgggttgcggtctt	*cidC*
cidCR	caattgcgatggcttgtcta	*cidC*
enoF	tcgcattagacggtactcca	*eno*
enoR	ccacgtgctactgcgataga	*eno*
lqoF	tttgggtggattccctatca	*lqo*
lqoR	ttggcatcgtgttgttcaat	*lqo*
PhiNM1F	ttgagtgaagcggaagttga	PhiNM1
PhiNM1R	tcgatttggtttgttcagca	PhiNM1
PhiNM2F	aaacgaaaattggaaaaagaaaa	PhiNM2
PhiNM2R	cgtactcctcttcgcactcc	PhiNM2

PhiNM3F	acaaccccaatcacaagagc	PhiNM3
PhiNM3R	ttggacattttgctgaggaac	PhiNM3
PhiNM4F	taacgagcgagtggcacata	PhiNM4
PhiNM4R	ccttgccaatctggtacgat	PhiNM4
sigHF	cggatgtataggtcggtcca	*sigH*
sigHR	tgaaaggctgctctgcacta	*sigH*
pfkAF	gggattgagggccttgtagt	*pfkA*
pfkAR	cattcctcactgatgcgttg	*pfkA*
pflBF	tgacggacgtccacttgtta	*pflB*
pflBR	agtttggttgtggagctgga	*pflB*
pgiF	atgatgattggtgctgcaaa	*pgi*
pgiR	tcgaattgtcgatattggt	*pgi*
pgmF	atgcaatggatcgtgacaaa	*pgm*
pgmR	gttggggcatcaaaattacg	*pgm*
pycAF	aaaacccatgggaacgactt	*pykA*
pycAR	gcgtttgaagcacgtaacaa	*pykA*
ald1F	ctggcattggttcaggattt	*ald1*
ald1R	gatcagcttcccatgcttgt	*ald1*
ald2F	gatcagcttcccatgcttgt	*ald2*
ald2R	actaggtggtgtcccaggag	*ald2*
glpDF	gccgaaactggtaaagaacg	*glpD*
glpDR	tgcattggtaaaagcatcca	*glpD*
citBF	gtgttttgttagaatctttacttg	*citB*
citBR	caagtaaagattctaacaaaagac	*citB*
sdhCF	catttttgctcgttcatttattagt	*sdhC*
sdhCR	actaataaatgaacggcaaaaatg	*sdhC*
16sF	gcggtaatacgtaggtggcag	*16s* RNA
16sR	tttccaatgaccctccacgg	*16s* RNA
sodAF	tcgtaaacaatgccagttaga	*sodA*
sodAR	taagcgtgttcccatacgtc	*sodA*
sodMF	cgcaacagttgaaggaacag	*sodM*
sodMR	caccgccattattacggact	*sodM*
glnAF	tggcgttagctgcaatctta	*glnA*
glnAR	ttcacgttcttcacacggttca	*glnA*
glnRF	taacggatttaacgccaagg	*glnR*
glnRR	ttccaaatcattgagtgagaaca	*glnR*
agrBF	cccattcctgtgtgcgacttat	*agrB*

agrFR	gaattgggcaaatggctctt	*agrB*
gprBF	cattttaaagtgaaaattggaggtg	*gprB*
gprRR	caaatcattggtaacgccataa	*gprB*
pyrAF	caggctgtaaaagcgagtcc	*pyrA*
pyrAR	tgaccgtttctccatcacaa	*pyrA*
capHF	gaggttgttggtggtgttcc	*capH*
capHR	gtttgtcaggcgttttctcc	*capH*
pyrGF	taacggatgatggtgcagaa	*pyrG*
pyrGR	cactttaccggctgtcacatt	*pyrG*
icaRF	aaatcgaactattcaattgatgc	*icaR*
icaRR	cagaaaattcctcaggcgta	*icaR*
glcAF	gctttgccacccatagcttct	*glcA*
glcAR	ggtggttagtcattcctgtaggtc	*glcA*
glcBF	gcgtgtgaacgatcccagttt	*glcB*
glcBR	gcaccagtactttatggaattcacg	*glcB*
glcCF	cacatctggatcaccaagttgagc	*glcC*
glcCR	cttaccgttttggcagacgg	*glcC*
glcUF	ggtgtagcacttacttcacttaaagct	*glcU*
glcUR	ggataggataaagccaccaattgcc	*glcU*
uhpTF	gtggtgccatagcaggtgg	*uhpT*
uhpTR	gctcttcccaaatttcttcagcacg	*uhpT*
gyrBF	ttagtgtgggaaattgtcgataat	*gyrB*
gyrBR	agtcttgtgacaatgcgtttaca	*gyrB*

Supplementary References

1 Fagerlund, A., Granum, P. E. & Havarstein, L. S. *Staphylococcus aureus* competence genes: mapping of the SigH, ComK1 and ComK2 regulons by transcriptome sequencing. *Mol Microbiol* **94**, 557-579, doi:10.1111/mmi.12767 (2014).

2 Yepes, A., Koch, G., Waldvogel, A., Garcia-Betancur, J. C. & Lopez, D. Reconstruction of mreB expression in *Staphylococcus aureus* via a collection of new integrative plasmids. *Appl Environ Microbiol* **80**, 3868-3878, doi:10.1128/AEM.00759-14 (2014).

3 Garcia-Betancur, J. C. *et al.* Cell differentiation defines acute and chronic infection cell types in Staphylococcus aureus. *Elife* **6**, doi:10.7554/eLife.28023 (2017).

4 O'Toole, G. A. & Kolter, R. Initiation of biofilm formation in *Pseudomonas fluorescens* WCS365 proceeds via multiple, convergent signalling pathways: a genetic analysis. *Mol Microbiol* **28**, 449-461 (1998).

5 van Sinderen, D., ten Berge, A., Hayema, B. J., Hamoen, L. & Venema, G. Molecular cloning and sequence of comK, a gene required for genetic competence in *Bacillus subtilis*. *Mol Microbiol* **11**, 695-703, doi:10.1111/j.1365-2958.1994.tb00347.x (1994).

6 van Sinderen, D. *et al.* comK encodes the competence transcription factor, the key regulatory protein for competence development in *Bacillus subtilis*. *Mol Microbiol* **15**, 455-462 (1995).

7 Hoa, T. T., Tortosa, P., Albano, M. & Dubnau, D. Rok (YkuW) regulates genetic competence in Bacillus subtilis by directly repressing comK. *Mol Microbiol* **43**, 15-26, doi:2727 [pii] (2002).

8 Branda, S. S., Gonzalez-Pastor, J. E., Ben-Yehuda, S., Losick, R. & Kolter, R. Fruiting body formation by *Bacillus subtilis*. *Proc Natl Acad Sci U S A* **98**, 11621-11626, doi:10.1073/pnas.19138419898/20/11621 [pii] (2001).

9 Kunst, F., T. Msadek & Rapoport, G. *Signal transduction network controlling degradative enzyme synthesis and competence in Bacillus subtilis*. (ASM Press, 1994).

10 Karimova, G., Pidoux, J., Ullmann, A. & Ladant, D. A bacterial two-hybrid system based on a reconstituted signal transduction pathway. *Proc Natl Acad Sci U S A* **95**, 5752-5756 (1998).

11 Feng, J. *et al.* Trapping and proteomic identification of cellular substrates of the ClpP protease in *Staphylococcus aureus*. *J Proteome Res* **12**, 547-558, doi:10.1021/pr300394r (2013).

12 Schlothauer, T., Mogk, A., Dougan, D. A., Bukau, B. & Turgay, K. MecA, an adaptor protein necessary for ClpC chaperone activity. *Proc Natl Acad Sci U S A* **100**, 2306-2311, doi:10.1073/pnas.0535717100 (2003).

13 Baba, T., Bae, T., Schneewind, O., Takeuchi, F. & Hiramatsu, K. Genome sequence of *Staphylococcus aureus* strain Newman and comparative analysis of staphylococcal genomes: polymorphism and evolution of two major pathogenicity islands. *J Bacteriol* **190**, 300-310, doi:JB.01000-07 [pii] 10.1128/JB.01000-07 (2008).

14 Thompson, J. D., Gibson, T. J., Plewniak, F., Jeanmougin, F. & Higgins, D. G. The CLUSTAL_X windows interface: flexible strategies for multiple sequence alignment aided by quality analysis tools. *Nucleic Acids Res* **25**, 4876-4882, doi:10.1093/nar/25.24.4876 (1997).

15 Morikawa, K. *et al.* Expression of a cryptic secondary sigma factor gene unveils natural competence for DNA transformation in *Staphylococcus aureus*. *PLoS Pathog* **8**, e1003003, doi:10.1371/journal.ppat.1003003 (2012).

16 Duthie, E. S. & Lorenz, L. L. Staphylococcal coagulase; mode of action and antigenicity. *J Gen Microbiol* **6**, 95-107 (1952).

17 Wermser, C. & Lopez, D. Identification of *Staphylococcus aureus* genes involved in the formation of structured macrocolonies. *Microbiology* **164**, 801-815, doi:10.1099/mic.0.000660 (2018).

18 Luong, T. T. & Lee, C. Y. The arl locus positively regulates *Staphylococcus aureus* type 5 capsule via an mgrA-dependent pathway. *Microbiology* **152**, 3123-3131, doi:10.1099/mic.0.29177-0 (2006).

19 Koch, G. *et al.* Evolution of resistance to a last-resort antibiotic in *Staphylococcus aureus* via bacterial competition. *Cell* **158**, 1060-1071, doi:10.1016/j.cell.2014.06.046 (2014).

20 Jelsbak, L. *et al.* The chaperone ClpX stimulates expression of *Staphylococcus aureus* protein A by Rot dependent and independent pathways. *PLoS One* **5**, e12752, doi:10.1371/journal.pone.0012752 (2010).

21 Oesterreich, B. *et al.* Characterization of the biological anti-staphylococcal functionality of hUK-66 IgG1, a humanized monoclonal antibody as substantial component for an immunotherapeutic approach. *Human vaccines & immunotherapeutics* **10**, 926-937 (2014).

22 Majerczyk, C. D. *et al. Staphylococcus aureus* CodY negatively regulates virulence gene expression. *J Bacteriol* **190**, 2257-2265, doi:JB.01545-07 [pii] 10.1128/JB.01545-07 (2008).

23 Novick, R. Properties of a cryptic high-frequency transducing phage in *Staphylococcus aureus*. *Virology* **33**, 155-166, doi:10.1016/0042-6822(67)90105-5 (1967).

24 Bae, T. *et al. Staphylococcus aureus* virulence genes identified by bursa aurealis mutagenesis and nematode killing. *Proc Natl Acad Sci U S A* **101**, 12312-12317, doi:10.1073/pnas.04047281010404728101 [pii] (2004).

25 Kornblum, J., Kreiswirth, B., Projan, S. J. Ross, H and Novick, R. P. in *Molecular biology of the staphylococci.* (ed R. P. Novick) (VCH Publishers, 1990).

26 Molina, A. *et al.* High prevalence in cystic fibrosis patients of multiresistant hospital-acquired methicillin-resistant *Staphylococcus aureus* ST228-SCCmecI capable of biofilm formation. *J Antimicrob Chemother* **62**, 961-967, doi:10.1093/jac/dkn302 (2008).

27 von Eiff, C. *et al.* A site-directed *Staphylococcus aureus* hemB mutant is a small-colony variant which persists intracellularly. *J Bacteriol* **179**, 4706-4712, doi:10.1128/jb.179.15.4706-4712.1997 (1997).

28 Burkholder, P. R. & Giles, N. H., Jr. Induced biochemical mutations in Bacillus subtilis. *Am J Bot* **34**, 345-348 (1947).

29 Vlamakis, H., Aguilar, C., Losick, R. & Kolter, R. Control of cell fate by the formation of an architecturally complex bacterial community. *Genes Dev* **22**, 945-953, doi:22/7/945 [pii] 10.1101/gad.1645008 (2008).

30 Kong, L. & Dubnau, D. Regulation of competence-specific gene expression by Mec-mediated protein-protein interaction in *Bacillus subtilis*. *Proc Natl Acad Sci U S A* **91**, 5793-5797, doi:10.1073/pnas.91.13.5793 (1994).
