# Supplementary material for: The induction of natural competence adapts staphylococcal metabolism to infection
Source: Nat Commun. 2022 Mar 21;13:1525. doi: 10.1038/s41467-022-29206-7 (PMC8938553; doi:10.1038/s41467-022-29206-7)
Supplement: Supplementary file 2 — Reporting Summary [file 41467_2022_29206_MOESM2_ESM.pdf]

nature portfolio

Corresponding author(s): Daniel Lopez

Last updated by author(s): Feb 28, 2022

Reporting Summary

Nature Portfolio wishes to improve the reproducibility of the work that we publish. This form provides structure for consistency and transparency in reporting. For further information on Nature Portfolio policies, see our Editorial Policies and the Editorial Policy Checklist.

Statistics

For all statistical analyses, confirm that the following items are present in the figure legend, table legend, main text, or Methods section.

n/a	Confirmed	
☐	☒	The exact sample size (n) for each experimental group/condition, given as a discrete number and unit of measurement
☐	☒	A statement on whether measurements were taken from distinct samples or whether the same sample was measured repeatedly
☐	☒	The statistical test(s) used AND whether they are one- or two-sided<br>*Only common tests should be described solely by name; describe more complex techniques in the Methods section.*
☒	☐	A description of all covariates tested
☐	☒	A description of any assumptions or corrections, such as tests of normality and adjustment for multiple comparisons
☐	☒	A full description of the statistical parameters including central tendency (e.g. means) or other basic estimates (e.g. regression coefficient) AND variation (e.g. standard deviation) or associated estimates of uncertainty (e.g. confidence intervals)
☐	☒	For null hypothesis testing, the test statistic (e.g. F, t, r) with confidence intervals, effect sizes, degrees of freedom and P value noted<br>*Give P values as exact values whenever suitable.*
☒	☐	For Bayesian analysis, information on the choice of priors and Markov chain Monte Carlo settings
☒	☐	For hierarchical and complex designs, identification of the appropriate level for tests and full reporting of outcomes
☒	☐	Estimates of effect sizes (e.g. Cohen's d, Pearson's r), indicating how they were calculated

Our web collection on statistics for biologists contains articles on many of the points above.

Software and code

Policy information about availability of computer code

Data collection	RNA DNA sequencing were performed using an Illumina HiSeq 2500 system in 2 × 100 bp paired-end read mode, according to the manufacturer's instructions.
Data analysis	For RNA seq analysis, Initial image analysis and base-calling were performed using HiSeq Control Software v.2.0.5 in combination with the RTA (real-tiFme analysis) v.1.17.20.0 program. CASAVA v.1.8.2 was used to generate and report run statistics and the final FASTQ files comprising the sequence information.<br><br>Fluorescence microscopy images were processed using the Leica Application Suite Advance Fluorescence Software.<br><br>Flow cytometry analyses were performed using FCAP Array Software v3.0 (BD Biosciences).<br><br>Statistical analyses were performed using GraphPad Prism v.7 Software

For manuscripts utilizing custom algorithms or software that are central to the research but not yet described in published literature, software must be made available to editors and reviewers. We strongly encourage code deposition in a community repository (e.g. GitHub). See the Nature Portfolio guidelines for submitting code & software for further information.

Data

Policy information about availability of data

All manuscripts must include a data availability statement. This statement should provide the following information, where applicable:

- Accession codes, unique identifiers, or web links for publicly available datasets
- A description of any restrictions on data availability
- For clinical datasets or third party data, please ensure that the statement adheres to our policy

Accession codes are are available in Material and Methods sections
The demultiplexed and coverage files that support the RNAseq and DNAseq findings were deposited in the NCBI's GEO database (GSE155016) https://www.ncbi.nlm.nih.gov/geo/query/acc.cgi?acc=GSE155016 and in the NCBI's SRA database (SRR12329121) https://www.ncbi.nlm.nih.gov/sra/?term=SRR12329121, respectively.

Field-specific reporting

Please select the one below that is the best fit for your research. If you are not sure, read the appropriate sections before making your selection.

☒ Life sciences ☐ Behavioural & social sciences ☐ Ecological, evolutionary & environmental sciences

For a reference copy of the document with all sections, see nature.com/documents/nr-reporting-summary-flat.pdf

Life sciences study design

All studies must disclose on these points even when the disclosure is negative.

Sample size	No statistical methods were used to predetermine sample size. The sample size was chosen to include at least 3 biologically independent experiments. Sample size was based on standard sample sizes from our past experiments and similarly to what is described for similar experiments in published articles (Koch et al., Cell 2014, 158:1060 and García-Fernández and Koch et al., Cell 2017, 171:1354)
Data exclusions	No data were excluded from the analyses
Replication	Experiments were independently performed at least 3 times and all attempts of replication were successful.
Randomization	Animals were randomly assigned to experimental groups. For in vitro experiments, cultured cells were uniformly plated, with random allocation of infection.
Blinding	The researchers performing RNA-seq and DNA-seq were blinded to strain selection. For mass-spectrometry analysis and EA-IRMS, the researchers performing sample preparation and analysis were were blinded to strain selection. For the remaining experiments, blinding was not applicable given the nature of the study. All critical experiments were repeated independently by at least two researchers.

Reporting for specific materials, systems and methods

We require information from authors about some types of materials, experimental systems and methods used in many studies. Here, indicate whether each material, system or method listed is relevant to your study. If you are not sure if a list item applies to your research, read the appropriate section before selecting a response.

Materials & experimental systems

n/a	Involved in the study	
☐	☒	Antibodies
☐	☒	Eukaryotic cell lines
☒	☐	Palaeontology and archaeology
☐	☒	Animals and other organisms
☒	☐	Human research participants
☒	☐	Clinical data
☒	☐	Dual use research of concern

Methods

n/a	Involved in the study	
☒	☐	ChIP-seq
☒	☐	Flow cytometry
☒	☐	MRI-based neuroimaging

Antibodies

Antibodies used	Rabbit-anti-FLAG (monoclonal, Sigma F1804) 1:5.000<br>Goat-anti-rabbit-HRP (BioRad 172-1019) 1:20.000<br>Rabbit-anti-6xHis tag polyclonal antibody (Rockland 600-401-382) 1:10000
Validation	We used commercial antibody reagents for Western-blot analysis. Therefore, relevant publications and/or validation results for each

antibody can be found through the RRID Portal (https://scicrunch.org/resources) or in the manusfacturer's website.

Eukaryotic cell lines

Policy information about cell lines

Cell line source(s)	A549 human lung epithelial cell line (ATCC-CCL-185)<br>Human monocyte-derived peripheral blood mononuclear cells (PBMCS) were isolated from buffy coat fraction.
Authentication	None of the cell lines was authenticated.
Mycoplasma contamination	All cells were tested and negative for Mycoplasma contamination
Commonly misidentified lines (See ICLAC register)	No commonly misidentified cell lines were used in this study.

Animals and other organisms

Policy information about studies involving animals; ARRIVE guidelines recommended for reporting animal research

Laboratory animals	Inbred 16-weeks old female mice BALB/c weighing 20 g to 24 g purchased from Charles River Laboratories.
Wild animals	No wild animals were involved.
Field-collected samples	No field-collected samples were involved.
Ethics oversight	Committee on Ethics in Animal Experiments of the Government of Lower Franconia (License number 55.2-2532-2-57)

Note that full information on the approval of the study protocol must also be provided in the manuscript.
