# Supplementary material for: The induction of natural competence adapts staphylococcal metabolism to infection
Source: Nat Commun. 2022 Mar 21;13:1525. doi: 10.1038/s41467-022-29206-7 (PMC8938553; doi:10.1038/s41467-022-29206-7)
Supplement: Supplementary file 4 — Description of Additional Supplementary Files [file 41467_2022_29206_MOESM4_ESM.pdf]

Title: Supplementary Data 1

Description: Down-regulated and up-regulated genes in the ∆comK mutant in comparison to WT strain and in the Kc strain in comparison to WT strain. LogFC represents fold-change gene expression. Description of gene function has been performed according to TIGRFAM and SEED function classification.
